# Conference Report: DATA ADMINISTRATION MANAGEMENT ASSOCIATION SYMPOSIUM Gaithersburg, MD May 17–18, 1994

**DOI:** 10.6028/jres.099.070

**Published:** 1994

**Authors:** Judith Newton

**Affiliations:** Information Systems Engineering Division, Computer Systems Laboratory, National Institute of Standards and Technology, Gaithersburg, MD 20899-0001

## 1. Introduction

Along with capital and human resources, an organization’s data represents one of its fundamental assets. Data administration (DA) attempts the effective planning, organization, and management of an enterprise’s data resource, with the intention of empowering the organization to achieve its mission and goals.

Achieving enterprise integration, and developing the supporting information technology infrastructure, is a critical need for organizations. Yet despite substantial attention by business managers, technologists, and vendors of tools and methodologies, there are few obvious solutions or guidance on how to accomplish this.

The Data Administration Management Association (DAMA) is the professional organization for data administrators. An international board over-sees a loose federation of local chapters in the United States, Canada, Australia, and Europe. The National Capital Region Chapter (NCR DAMA) has monthly meetings from September through April, as well as a Symposium in May.

NCR DAMA held its seventh annual Symposium at NIST on May 17–18, 1994. The theme this year was *Enterprise Integration in the Turbulent 90s.* Attended by over 200 Federal and private industry data administrators, the Symposium was cosponsored by NIST and NCR DAMA.

The Symposium emphasized the practices, technologies, activities, initiatives and ideas that deliver clearly visible value to the users, or “customers” of data administration. In addition to presentations by nationally recognized experts and practitioners, it included breakout dialog sessions and panel discussions. Topics ranged from the keynote speech on the National Information Infrastructure to the latest implementation of the Information Resource Dictionary System (IRDS) standard. New this year was a Vendor Exhibit Area, where the latest tools for implementing the practices of Data Administration could been seen.

## 2. Speakers

The keynote speaker was Arati Prabhakar, Director of NIST, describing the government’s role in the National Information Infrastructure.

Mary “Bunny” Smith provided a personalized perspective of information systems design for new, very small businesses. Spreadsheets are invaluable both for planning and cost accounting. One can not start to model one’s business too soon, even if it occurs on the kitchen table.

Dr. Mike Mestrovitch, DoD, discussed transforming the enterprise through Enterprise Integration, and what transformation is planned for DoD. It will involve changing every aspect of the organization to meet new circumstances and expectations. It is a way of using information as a strategic asset to manage the enterprise far more effectively and efficiently. It bridges functional and technical boundaries to increase flexibility and to focus all available capabilities on mission results. The process includes: establishing a vision for the future; creating a sense of urgency about the vision; redefining the business processes; redefining resource capabilities; creating a new work environment; re-creating management systems and structures; and building an information and technology architecture to empower the organization to execute.

The last speaker, John Zachman of Zachman International, presented his Information Architecture concept together with his personal view of the future of manufacturing and technology. We must change from Custom-design-and-build and Provide- from-stock to Assemble-to-order processes. He issued a challenge for everyone in the audience to rise to meet “The New Realities of the Information Age.”

## 3. Concurrent Sessions

A series of concurrent sessions focused on various managerial and technical aspects of enterprise integration. Several sessions addressed the National Performance Review. Others considered data modeling, EDI, and open systems.

## 4. Panels

The following panel discussions were presented:

- Repository, A Missing Link in Enterprise Integration, Carla von Bernewitz, DoD, moderator;
- Using IDEF in Conjunction with Information Engineering, Vince Cordovano, James Martin Consulting, moderator;
- Standards for Data Administration, Judith J. Newton, NIST, moderator;
- GOV-SIG: Stepping Stone to Public Sector Integration, Pam Piper, DoD, and Tom Kurihara, NIST, moderators;
- Linking IRM Initiatives to Strategic Planning, Stephanie Wietecha, FC Business Systems, moderator;
- Reengineering a Data Management Program, Patricia Simes, SRA, moderator.

## 5. Proceedings

The proceedings of this, the Third, the Fifth, and the Sixth Symposia were distributed at the event. The Proceedings of the First,^1^ Second,^2^ and Fourth^3^ Symposia were published by NIST and copies are still available. The Eighth Annual Symposium will be held May 16–17,1995.

## Footnotes

1 Judith J. Newton, and Frankie E. Spielman, eds., Data Administration: Management and Practice, Proceedings of the First DAMA Symposium, NIST Special Publication 500-159, National Institute of Standards and Technology, Gaithersburg, MD, October 1988.

2 Judith J. Newton, and Frankie E. Spielman, eds.. Data Administration: Standards and Techniques, Proceedings of the Second Annual DAMA Symposium, NISTIR 90-4292, National Institute of Standards and Technology, Gaithersburg, MD, April 1990

3 Judith J. Newton, Mary Lou Melley, and Holly Higgins, eds., Managing Data: From Vision to Reality, Proceedings of the Fourth Annual DAMA Symposium, NISTIR 4843, National Institute of Standards and Technology, Gaithersburg, MD, May 1992.

